# Co-Production of Isoprene and Lactate by Engineered *Escherichia coli* in Microaerobic Conditions

**DOI:** 10.3390/molecules26237173

**Published:** 2021-11-26

**Authors:** Tao Cheng, Xiuhong Liang, Yaqun Wang, Ningning Chen, Dexin Feng, Fengbing Liang, Congxia Xie, Tao Liu, Huibin Zou

**Affiliations:** 1 State Key Laboratory Base of Eco-Chemical Engineering, College of Chemical Engineering, Qingdao University of Science and Technology, Qingdao 266042, China; chengtao@qibebt.ac.cn (T.C.); liangxiuhong@mails.qust.edu.cn (X.L.); yaqunwanghsh@163.com (Y.W.); chennngust@163.com (N.C.); fengdx@qibebt.ac.cn (D.F.); taoyanghe@hotmail.com (T.L.); 2 CAS Key Laboratory of Bio-Based Materials, Qingdao Institute of Bioenergy and Bioprocess Technology, Chinese Academy of Sciences, Qingdao 266101, China; liangfb@qibebt.ac.cn; 3 State Key Laboratory Base of Eco-Chemical Engineering, College of Chemistry and Molecular Engineering, Qingdao University of Science and Technology, Qingdao 266042, China

**Keywords:** lactic acid, isoprene, mevalonate pathway, co-production, microaerobic fermentation

## Abstract

Lactate and isoprene are two common monomers for the industrial production of polyesters and synthetic rubbers. The present study tested the co-production of D-lactate and isoprene by engineered *Escherichia coli* in microaerobic conditions. The deletion of alcohol dehydrogenase (*adhE*) and acetate kinase (*ackA*) genes, along with the supplementation with betaine, improved the co-production of lactate and isoprene from the substrates of glucose and mevalonate. In fed-batch studies, microaerobic fermentation significantly improved the isoprene concentration in fermentation outlet gas (average 0.021 g/L), compared with fermentation under aerobic conditions (average 0.0009 g/L). The final production of D-lactate and isoprene can reach 44.0 g/L and 3.2 g/L, respectively, through fed-batch microaerobic fermentation. Our study demonstrated a dual-phase production strategy in the co-production of isoprene (gas phase) and lactate (liquid phase). The increased concentration of gas-phase isoprene could benefit the downstream process and decrease the production cost to collect and purify the bio-isoprene from the fermentation outlet gas. The proposed microaerobic process can potentially be applied in the production of other volatile bioproducts to benefit the downstream purification process.

## 1. Introduction

With respect to the exhaustion of fossil fuels and increasing environmental issues, biopolymers have recently been developed as alternatives to fossil-fuel-derived synthetic polymers. With the rapid development of advanced biotechnologies, variable bio-monomers can be microbially produced from renewable biomass [1]. Isoprene and lactic acid are two representative bio-monomers that can be biosynthesized and further applied in the preparation of green polymers [2,3].

Variable strains of lactic acid bacteria (LAB) are commonly used for the industrial production of monomer-grade lactic acid [4]. Engineered strains have been developed for lactate fermentation, such as the engineered *Escherichia coli* [5] and *Klebsiella pneumoniae* [6]. Lactate fermentation is normally performed under anaerobic and microaerobic conditions [7]. Engineered *E. coli* strains have also been utilized in the biosynthesis of isoprene through the mevalonate (MVA) or 1-deoxy-D-xylulose-5-phosphate (DXP) pathways [8]. Unlike lactate fermentation, isoprene fermentation is normally controlled under aerobic conditions [9,10]. In the aerobic fermentation process, compressed air is pumped to supply oxygen and eject isoprene, and the concentration of isoprene is below 2% in the vapor phase—far less than the concentration of petroleum-isoprene (10–20%) in the extractive distillation process [2]. The low concentration of bio-isoprene produced in the aerobic fermentation process impedes the recovery efficiency in the downstream process. However, the fermentation of isoprene under anaerobic and microaerobic conditions has not been well studied—especially for engineered *E. coli* strains, which are usually utilized in the microbial production of isoprene and isoprenoids. 

**Figure 1:**
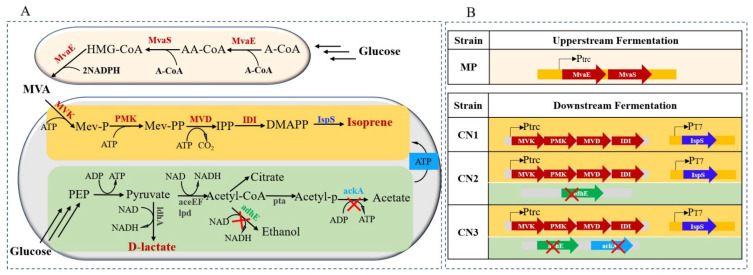
The metabolic pathway and strain information for the co-production of isoprene and lactate: (**A**) Mevalonate (MVA) is produced by the upstream fermentation from glucose; isoprene and lactate are co-produced by the downstream fermentation from MVA and glucose substrates. (**B**) Construction of the co-production strains carrying the downstream genes of the MVA pathway and deleting the genes of *ackA* and *adhE*. Enzymes involved in this pathway—MvaE: acetyl-CoA acetyltransferase/HMG-CoA reductase; MvaS: HMG-CoA synthase; MVK: mevalonate kinase; PMK: phosphomevalonate kinase; MVD: mevalonate pyrophosphate decarboxylase; IDI: IPP isomerase; IspS: isoprene synthase; aceEF: pyruvate dehydrogenase; lpd: lipoamide dehydrogenase; adhE: alcohol/aldehyde dehydrogenase; pta: phosphate acetyltransferase; ackA: acetate kinase. Pathway intermediates—A-CoA: acetyl-CoA; AA-CoA: acetoacetyl-CoA; HMG-CoA: 3-hydroxy-3-methylglutaryl-CoA; Mev-P: mevalonate 5-phosphate; Mev-PP: mevalonate 5-diphosphate; IPP: isopentenyl pyrophosphate; DMAPP: dimethylallyl diphosphate; PEP: phosphoenolpyruvate; Acetyl-p: acetyl phosphate; NADH: nicotinamide adenine dinucleotide; NAD: nicotinamide adenine dinucleotide; ATP: adenosine triphosphate: ADP: adenosine diphosphate; pTrc: Trc promoter; pT7: T7 promoter.

**Figure 2:**
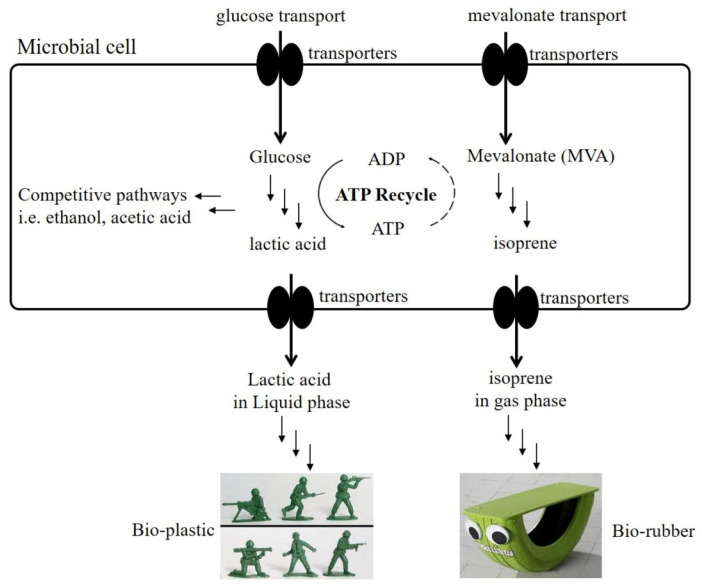
The routine method of the co-production of isoprene and lactic acid. In the microbial cell, the lactic acid and isoprene were produced using the substrates of glucose and mevalonate, respectively, in dual phase and under microaerobic fermentation conditions.

Different biotechnologies and strategies have been developed to facilitate the downstream separation process after fermentation [8]. The present study aimed to research the microaerobic process for the biosynthesis of isoprene, and to compare it with the aerobic process, which is routinely used in isoprene fermentation (Figure 1). In addition, this study tested the co-production of isoprene and lactic acid under microaerobic conditions. During the fermentation process, the presence of isoprene and lactic acid in dual phases (isoprene in gas, lactic acid in broth) could achieve technical and economic benefits for the downstream process (Figure 2).

## 2. Results and Discussion 

### 2.1. The Deletion of AdhE and AckA Improved the Lactate Production of Isoprene-Producing Strains

**Figure 3:**
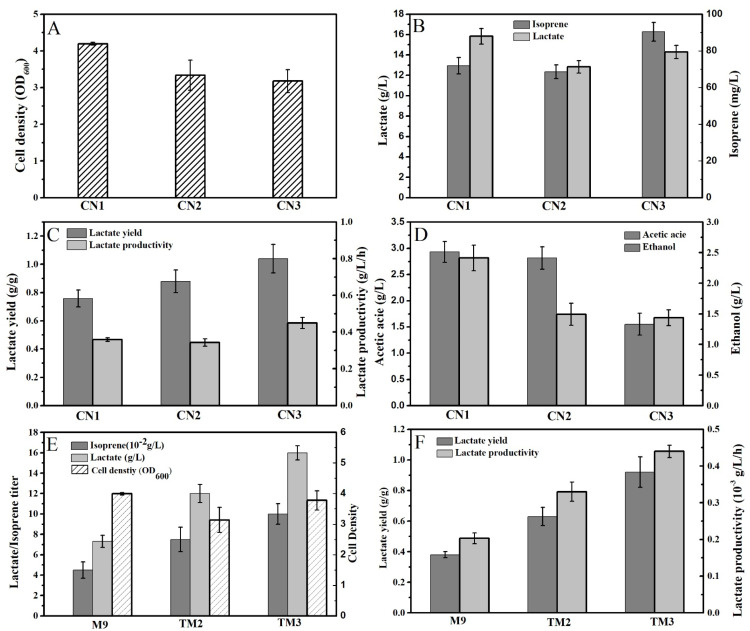
(**A**–**D**) Comparison of the levels of isoprene, lactate, acetic acid, and ethanol in flask fermentation by *E. coli* CN1, CN2, and CN3. (**E**,**F**) Levels of isoprene and lactate in flask fermentation by *E. coli* CN3 in different media are compared. (**A**) Cell density after 48 h of fermentation in M9 medium; (**B**) lactate and isoprene titers after 48 h of fermentation in M9 medium; (**C**) lactate yield and productivity after 48 h of fermentation in M9 medium; (**D**) acetate and ethanol titers after 48 h of fermentation in M9 medium; (**E**) the titers of isoprene and lactate after 40 h of fermentation in different media by CN3; (**F**) lactate yield and productivity after 40 h of fermentation in different media by CN3. The data shown are means of three parallel replicates, and the error bars present their standard deviation.

For the preliminary investigation of the co-production of isoprene and lactate by different strains, flask-level experiments (48 h of fermentation in M9 medium) were performed to compare the production of isoprene, lactate, and their byproducts (ethanol and acetic acid) by different strains. As shown in Figure 3A–D, CN2 (deletion of *adhE*) presented decreased production of alcohol compared to that of CN1, but did not present increased production of lactate. CN3 (deletion of both *adhE* and *ackA*) presented higher production of lactate than that of CN1 and CN2 (Figure 3B). 

The results demonstrate that the deletion of *adhE* and *ackA* can reduce the production of ethanol/acetate and improve the production of lactate by the isoprene-producing strain CN3. Similar results have also been shown in other lactate-producing *E. coli* strains, such as *E. coli* CICIM B0013-070 [11] and *E. coli* SZs [12]. 

This study further analyzed the optical purity of lactate, and confirmed that only D-lactate is produced by CN1, CN2, and CN3. Similar results have also been shown in other *E. coli* strains with the native D-lactate dehydrogenase (LDH, encoded by the *ldhA* gene) for D-lactate fermentation [12].

### 2.2. Medium Optimization Improved the Co-Production of Isoprene and D-Lactate by CN3

Based on the CN3 strain, the study further tested flask production of isoprene and D-lactate with different media. After 40 h of fermentation with M9 medium, CN3 could produce around 0.04 g/L isoprene and 7.4 g/L D-lactate, while the corresponding yield was 0.38g/g at almost 36 h after induction. When M9 medium was replaced with TM2 medium, the isoprene production was doubled to around 0.08 g/L, and the production of D-lactate was increased to 12.2 g/L (yield 0.6 g/g). The highest levels of isoprene (around 0.1 g/L) and D-lactate (titer 16.1 g/L, yield 0.9 g/g) were found in flask fermentation in TM3 medium (Figure 3E,F).

The results demonstrated that the production of both isoprene and lactate was positively affected by the addition of organic supplementation in the fermentation medium. Compared with the minimum medium M9, more organic nutrients (yeast extract or beef extract) are present in the TM2 and TM3 media, making them beneficial for the co-production of isoprene and lactate by engineered *E. coli*.

As shown in Figure 3E, the addition of betaine in TM3 medium further improved the production of both lactate and isoprene. It is speculated that the addition of betaine might contribute to protecting the microbial cells and the cellular enzymes against osmotic stresses (high concentrations of soluble products and substrates), similar to studies on the fermentation of mevalonate, lactate, ethanol, lysine, and pyruvate [5,13,14,15].

### 2.3. Fed-Batch Fermentation of Lactate and Isoprene under Aerobic or Microaerobic Conditions

The above shake-flask experiments showed that CN3 presented higher production in the co-fermentation of D-lactate and isoprene, and that supplementation with organic nutrients and betaine could improve the co-production of D-lactate and isoprene. To further study the fermenter-based fermentation of isoprene and lactate, fed-batch fermentation by CN3 was tested in a 5 L fermenter under aerobic or microaerobic conditions (Figure 1).

**Figure 4:**
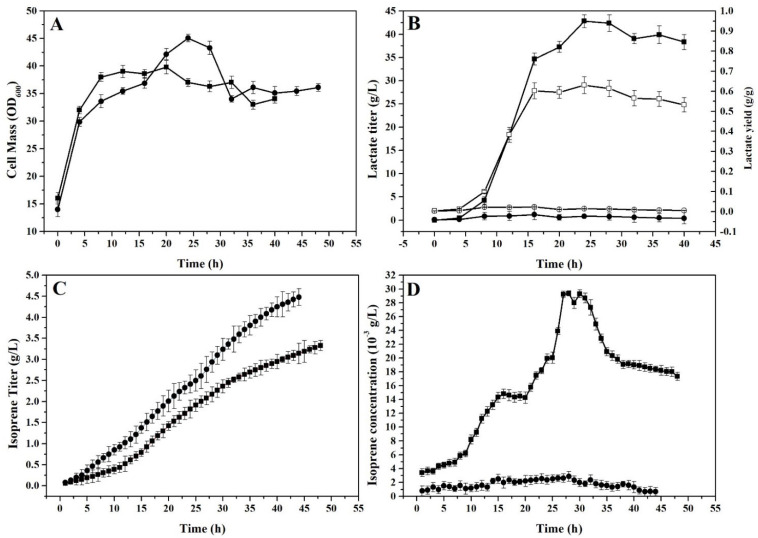
Comparison of the co-production of isoprene and lactate under aerobic and microaerobic conditions in fed-batch fermentation by *E. coli* CN3: (**A**) Profiles of cell density, (**B**) lactate titer and yield, (**C**) isoprene titer, and (**D**) isoprene concentration in outlet gas. Values of tested samples under aerobic conditions are shown by round dots (●), titers of tested samples under microaerobic conditions are shown by square dots (■), lactate yield under aerobic and microaerobic conditions are shown by hollow circles (○) and hollow squares (□), respectively. The data shown are the means of three parallel replicates, and the error bars represent their standard deviation.

As shown in Figure 4C, the total isoprene titer reached 4.5 g/L under aerobic conditions, which was higher than the total isoprene titer of 3.2 g/L under microaerobic conditions. The increased isoprene production corresponded with better cell growth under aerobic conditions (Figure 4A). These results are consistent with a previous study showing that aerobic conditions offer higher biomass density for *E. coli* [16]. Under aerobic conditions, more ATP was generated through the respiratory chain and the oxidative phosphorylation system, providing a fast growth rate, high biomass density, and high productivity [16].

Although the total production of isoprene was decreased by 30% under microaerobic conditions, the titer of D-lactate and the isoprene concentration in the outlet gas were improved. The lactate titer was increased from 2.0 g/L to 44.0 g/L (22-fold), and the maximum yield of lactate reached 0.58 g/g (Figure 4B). In addition, the average isoprene concentration in the outlet gas was around 0.021 g/L, and its maximum concentration could reach 0.03 g/L under microaerobic conditions (Figure 4D), which is significantly higher (34-fold increase) than that under aerobic conditions (average 0.0009 g/L in the outlet gas). The production of isoprene in this study was lower than previously reported [17] when employing exogenous MVA and homologous MEP pathways to convert glucose to isoprene via engineered *E. coli*. However, the high concentration of isoprene in the exhaust during the fermentation process has not been reported in previous studies. According to a previous report finding that a 10-fold increase in isoprene concentration will decrease the purification cost of bio-isoprene by 20% [2], the downstream processing cost should decrease by 60% compared to the aerobic process.

The above results indicate that the microaerobic conditions were beneficial for the co-production of isoprene and lactate, and that the limited dissolved oxygen environment caused by a low gas flow rate was conducive to inducing the co-fermentation of lactate. *E. coli* CN3 can utilize alternative electron receptors (e.g., formate nitrate and nitrite) instead of oxygen to survive, and can maintain its redox balance under microaerobic conditions, similar to the previous study [17]. Moreover, there are several advantages of the fermentation process under microaerobic conditions, including easier reactor design and control—beneficial for the co-production of multiple products [18]. 

In this study, the improved concentration of isoprene in fermentation outlet gas offers a promising advantage: it is beneficial for the downstream collection and purification of bio-isoprene. As a non-polar volatile organic gas, it is difficult to collect low-concentration isoprene from outlet gas, and the improved isoprene concentration in outlet gas can significantly decrease the downstream cost [2]. The microaerobic process has another advantage for the real-time monitoring of the fermentation process, as the dissolved oxygen level is kept at a low and consistent level compared with the fluctuated DO level in aerobic conditions. According to Industry 4.0 [19], it is feasible for microaerobic processes to utilize automatic real-time monitoring and control techniques in order to optimize the general fermentation process. 

## 3. Material and Methods

### 3.1. Strain Engineering

All strains, plasmids, and primers used in this study are summarized in Table 1. The engineered strain of CM1 (BL21(DE3)::Trc-low), which has four genes of the lower MVA pathway with the promoter of Trc on sites of the *glmS* and *glmU* genes, was used as the parent strain for engineering lactate- and isoprene-producing strains [20]. *E. coli* DH5α (TaKaRa, Beijing, China) was used for cloning and plasmid storage. *E. coli* χ7213 was used to construct a suicide vector. The plasmids of pYJM4 and pYJM16 were constructed in a previous study [21]. pYJM4 carries the gene of isoprene synthase (IspS). pYJM16 carries four genes of the downstream process of the MVA pathway: mevalonate-5-kinase (MVK), mevalonate-3-phosphate-5-kinase (PMK), mevalonate-5-pyrophosphate decarboxylase (MVD), and isopentenyl pyrophosphate isomerase (IDI) from *Saccharomyces cerevisiae* (Table 1). 

The suicide vector (pRE112)-mediated method [22] was utilized for genome editing in this study. This study first engineered CM2 with the *adhE* gene removed. Firstly, the flanking regions of the *adhE* gene were amplified with PrimeSTAR Max DNA Polymerase (TaKaRa, Beijing, China) and ligated using overlap extension PCR to generate the homologous arms, which were then subcloned into pRE112 to generate the pRE112-ΔadhE (Table 1). The plasmid of pRE112-ΔadhE was employed in the suicide-vector-mediated approach described previously [20], producing CM2, which deleted the *adhE* gene and carried the low MVA pathway on the chromosome. 

A similar genome editing strategy was applied to generate CM3 from CM2 (further deleting the genes of *adhE* and *ackA*). The plasmid of pYJM8 was transformed to CM1, CM2, and CM3 to obtain CN1, CN2, and CN3, respectively, for isoprene and lactate fermentation.

### 3.2. MVA Fermentation and Purification

The MVA fermentation and purification were performed as described previously [15]. Fermentation was performed in a 5 L bioreactor (Applikon Biotechnology, ez-control, Netherlands) containing 3 L of TM3 medium. A single MP strain colony was cultivated in 100 mL of LB medium in a 250 mL shake flask and shaken at 200 rpm for 8 h at 37 °C, before being transferred into a fresh MVA fermentation medium [15] with the amount of inoculum at 5%. The fermentation process was carried out under the following conditions: the temperature was 32 °C, the pH was maintained at 7.0 via automatic addition of ammonia water, and the dissolved oxygen level was maintained at 30% saturation by adjusting the stirring rate (400–800 rpm) at a constant airflow of 1.0 vessel volumes per minute (vvm). Glucose (50%) was fed into the bioreactor at an appropriate rate to maintain the residual glucose in the culture broth below 0.5 g/L. When the cell density reached 15 of OD_600_, 0.5 mM IPTG was added to the culture broth.

Following MVA fermentation, the culture broth was centrifuged at 8000–10,000 rpm for 10 min to collect the supernatant. To convert MVA to mevalonolactone, the supernatant was adjusted to a pH of 2.0 with 3 M HCl and incubated at 45 °C for 1 h. The solution was saturated with Na_2_SO_4_ and then extracted with an equivalent amount of ethyl acetate. The top organic phase produced by centrifugation was then evaporated to mevalonolactone using vacuum rotary evaporation. Mevalonolactone was neutralized to pH 7.0 with 1 M NaOH, and then converted to mevalonate for use as a feeding precursor for isoprene and lactate fermentation.

### 3.3. Co-Fermentation of Isoprene and Lactic Acid at Flask Levels

Single clones of different strains (CN1, CN2, or CN3) were grown overnight in 5 mL of LB medium with the appropriate antibiotic at 37 °C and shaken at 180 rpm. Then, 100 mL of different media (M9, TM2, or TM3) in a 500 mL saline bottle were inoculated with 1 mL of each culture and shaken at 180 rpm for 7 h. The strains were induced with 0.5 mmol IPTG when the OD_600_ reached around 0.6~0.8. After induction, the flasks were supplemented with mevalonate to final concentrations of 2 g/L and sealed with a rubber plug to form the microaerobic environment in order to facilitate the formation of lactic acid and isoprene formation. The cell mass and levels of isoprene, lactate, acetic acid, and ethanol were determined after incubation for 40 h or 48 h at 30 °C, 180 rpm.

TM2 contains 2.1 g/L critic acid monohydrate, 9.8 g/L K_2_HPO_4_·3H_2_O, 0.3 g/L ferric ammonium citrate, 0.5 g/L yeast extract, 20 g/L glucose, and 0.24 g/L MgSO_4_. TM3 medium contains 3 g/L (NH_4_)_2_SO_4_, 2.5 g/L KH_2_PO_4_, 0.24 g/L MgSO_4_·7H_2_O, 1.86 g/L KCl, 1 g/L sodium citrate, 1 g/L citric acid, 1g/L betaine, 20 g/L glucose, and 5 g/L beef extract. All media also contain 34 μg/mL chloramphenicol and 1 mL/L storage solution of trace elements (each 100 mL storage solution of trace elements containing 2.47 g H_3_BO_3_, 1.58 g MnCl_2_·4H_2_O, 0.37 g (NH_4_)_6_Mo_7_O_24_·4H_2_O, 0.29 g ZnSO_4_·7H_2_O, and 0.25 g CuSO_4_·5H_2_O). All of the reagents were purchased from Sangong (Sangong Group, Qingdao, China).

### 3.4. Fed-Fermentation of Isoprene and Lactate

A single colony of CN3 was inoculated into 3 mL of LB medium and shaken for 6 h at 37 °C and 200 rpm. Then, 100 mL of M9 minimal medium containing the appropriate antibiotic in a 500 mL shake flask was inoculated with 1 mL of the culture and cultivated at 37 °C and 200 rpm for 8 h. Following that, the culture was transferred to a 5 L fermenter with 3 L of TM3 medium with a 3% inoculum. The fermentation process was carried out under the following conditions: the pH was maintained at 7.0 by the automated addition of ammonia water, and the temperature was maintained at 32 °C. After the initial glucose (20 g/L) was consumed, a solution comprising 60% (*v*/*v*) glucose and 30 g/L of mevalonate was fed into the fermenter at an appropriate rate in order to maintain the residual glucose concentration of roughly 5 g/L. When the cell density reached 12 of OD_600_, 0.25mM IPTG was added to the culture broth. For aerobic conditions, the dissolved oxygen level was maintained at 30% saturation by adjusting the stirring rate at 400–800 rpm (revolutions per minute) while maintaining a constant airflow of 1.0 vvm. For microaerobic conditions, the aeration rate was reduced from 1.0 vvm to 0.1 vvm after 4 h of induction, and the dissolved oxygen level was maintained at 0% by adjusting the stirring rate. After 8 h of induction, 20 g/L of calcium carbonate was added to the fermenter. Cell density and the production of lactate were monitored by periodic sampling. In order to accurately assess the production of isoprene in the fermentation process, 1 mL of outlet gas was drawn every 30 min to detect the concentration of isoprene.

### 3.5. Analytical and Statistical Methods

The intermediate metabolites of lactate, acetic acid, ethanol, and mevalonate were determined using liquid chromatography (Agilent Technologies, Inc, Agilent 1200, Santa Clara, CA, USA) coupled with an Aminex HPX-87H column (Bio-Rad Laboratories, Inc., Hercules, CA, USA) and a differential refractive index detector (Agilent Technologies Inc, Santa Clara, CA, USA). The column temperature was maintained at 50 °C, with the mobile phase containing 5 mM H_2_SO_4_, and a flow rate of 0.5 mL/min.

For the isoprene assay, 1 mL of gas was collected and analyzed using GC, as described previously [15]. 

Three replicated fermentations were performed, and the data of mean values and standard deviations were recorded. The significance of differences between mean values of different testing groups was compared using Student’s *t*-test.

## 4. Conclusions

The present study demonstrates a microaerobic process in the fermentation of both lactate and isoprene. The proposed process will benefit the downstream separation process, as (1) both products are produced in dual phase during fermentation, and can be separately purified, and (2) although the total isoprene production was slightly decreased, microaerobic fermentation significantly improved the isoprene concentration in the outlet gas, benefiting the downstream purification process. The co-production method and microaerobic process may be applied in the bioproduction of other volatile products in future studies.

## Figures and Tables

**Table 1:** Strains, plasmids, and primers used in this study.

Strains/Plasmids/Prmers	Description	Source
strains		
*E.coli* BL21(DE3)	F-ompT hsdSB(rB-mB-) gal dcm rne131(DE3)	Invitrogen
*E.coli* DH5	Cloning host	Invitrogen
*E.coli* χ7213	Host strain for pRE112, DAP auxotrophic strain	[20]
MP	BL21(DE3)/pYJM16	[9]
CM1	BL21(DE3)::ptrc-mvk-pmk-mvd-idi	[20]
CM2	BL21(DE3)::ptrc-mvk-pmk-mvd-idi△adhE	This study
CM3	BL21(DE3)::ptrc-mvk-pmk-mvd-idi△adhE△ackA	This study
CN1	CM1/pYJM8	This study
CN2	CM2/pYJM8	This study
CN3	CM3/pYJM8	This study
Plasmids		
pRE112	Suicide vector, R6K origin, chloramphenicol resistant	[22]
pRE112-ΔSU	pRE112 derivative carrying genes glmS, glmU	This study
pRE112-ΔSU-trc-low	pRE112 derivative carrying genes glmS, glmU, ERG8, ERG12, ERG19 and IDI1, Trc promoter	This study
pRE112-adhE	Suicide vector for construction of △adhE mutant	This study
pRE112-ackA	Suicide vector for construction of △ackA mutant	This study
pYJM8	pACYCDuet-1 derivative carrying isoprene synthase gene ispS, T7 promoter, CmR	[9]
pYJM14	pTrcHis2B derivative carrying phosphomevalonate kinase gene ERG8, mevalonate kinase gene ERG12, mevalonate pyrophosphate decarboxylase gene ERG19 and IPP isomerase gene IDI1, Trc promoter, Ap^R^	[9]
pYJM16	pACYCDuet-1 derivative carrying acetyl-CoA acetyltransferase/hydroxymethylglutaryl-CoA (HMG-CoA) reductase gene mvaE and HMG-CoA synthase gene mvaS, T7 promoter, CmR	[9]
primers		
adhE_F_F′	CGAGTACTCCACAGACAGGTTGGCTGTAAG	
adhE_F_R	GTAGGTATCCAGATCTTCGACGATACCCATGC	
adhE_R_F	TATCGTCGAAGATCTGGATACCTACTACGGTCGTG	
adhE_R_R	GCTCTAGAGATGAGATTCGTTCGGAACAT	
ackA_F_F	CTAGTACTGATAACAGAACGATTATCCG	
ackA_F_R	TATATACGGCAGAAATTGATGATCG	
ackA_R_F	CATAAAACGGATCGCATAACGC	
ackA_R_R	GCTCTAGACATAACGAAGACGATTTCCGC	

Note: The restriction sites in the primers were underlined.

## Data Availability

No applicable.

## Sample Availability

Strains are available from the authors.

## References

[1] Kawaguchi H., Ogino C., Kondo A. (2017). Microbial conversion of biomass into bio-based polymers. Bioresour. Technol..

[2] Whited G., Feher F., Benko D., Cervin M., Chotani G., McAuliffe J., LaDuca R., Ben-Shoshan E., Sanford K. (2010). Development of a gas-phase bioprocess for isoprene-monomer production using metabolic pathway engineering. Ind. Biotechnol..

[3] Park S.J., Kim T.W., Kim M.K., Lee S.Y., Lim S.C. (2012). Advanced bacterial polyhydroxyalkanoates: Towards a versatile and sustainable platform for unnatural tailor-made polyesters. Biotechnol. Adv..

[4] John R.P., Nampoothiri K.M., Pandey A. (2007). Fermentative production of lactic acid from biomass: An overview on process developments and future perspectives. Appl. Microbiol. Biotechnol..

[5] Zou H., Wu Z., Xian M., Liu H., Cheng T., Cao Y. (2013). Not only osmoprotectant: Betaine increased lactate dehydrogenase activity and L-lactate production in lactobacilli. Bioresour. Technol..

[6] Feng X.J., Ding Y.M., Xian M., Xu X., Zhang R.B., Zhao G. (2014). Production of optically pure D-lactate from glycerol by engineered Klebsiella pneumoniae strain. Bioresour. Technol..

[7] Zhou S.D., Shanmugam K.T., Ingram L.O. (2003). Functional replacement of the *Escherichia coli* d-(-)-lactate dehydrogenase gene (ldhA) with the l-(+)-lactate dehydrogenase gene (ldhL) from Pediococcus acidilactici. Appl. Environ. Microb..

[8] Wang C.L., Zada B., Wei G.Y., Kim S.W. (2017). Metabolic engineering and synthetic biology approaches driving isoprenoid production in *Escherichia coli*. Bioresour. Technol..

[9] Yang J., Xian M., Su S., Zhao G., Nie Q., Jiang X., Zheng Y., Liu W. (2012). Enhancing production of bio-isoprene using hybrid MVA pathway and isoprene synthase in *E. coli*. PLoS ONE.

[10] Yang C., Gao X., Jiang Y., Sun B., Gao F., Yang S. (2016). Synergy between methylerythritol phosphate pathway and mevalonate pathway for isoprene production in Escherichia coli. Metab. Eng..

[11] Zhou L., Niu D.D., Tian K.M., Chen X.Z., Prior B.A., Shen W., Shi G.Y., Singh S., Wang Z.X. (2012). Genetically switched D-lactate production in Escherichia coli. Metab. Eng..

[12] Zhou S., Grabar T.B., Shanmugam K.T., Ingram L.O. (2006). Betaine tripled the volumetric productivity of d(-)-lactate by *Escherichia coli* strain SZ132 in mineral salts medium. Biotechnol. Lett..

[13] Xu J., Xia X., Zhang J., Guo Y., Zhang W. (2014). An overlooked effect of glycine betaine on fermentation: Prevents caramelization and increases the L-lysine production. J. Microbiol. Biotechnol..

[14] Zou H.B., Chen N.N., Shi M.X., Xian M., Song Y.M., Liu J.H. (2016). The metabolism and biotechnological application of betaine in microorganism. Appl. Microbiol. Biot..

[15] Liu H., Cheng T., Zou H.B., Zhang H.B., Xu X., Sun C., Aboulnaga E., Cheng Z.K., Zhao G., Xian M. (2017). High titer mevalonate fermentation and its feeding as a building block for isoprenoids (isoprene and sabinene) production in engineered *Escherichia coli*. Process. Biochem..

[16] Clark D.P. (1989). The Fermentation Pathways of *Escherichia coli*. FEMS Microbiol. Lett..

[17] Partridge J.D., Sanguinetti G., Dibden D.P., Roberts R.E., Poole R.K., Green J. (2007). Transition of *Escherichia coli* from aerobic to micro-aerobic conditions involves fast and slow reacting regulatory components. J. Biol. Chem..

[18] Lange J., Takors R., Blombach B. (2017). Zero-growth bioprocesses: A challenge for microbial production strains and bioprocess engineering. Eng. Life Sci..

[19] Lopez P.C. (2020). Towards Industry 4.0 in the Bioprocessing Industries: ‘Real-Time’ monitoring and Control of Lignocellulosic Ethanol Fermentations. Ph.D. Thesis.

[20] Cheng T., Zhao G., Xian M., Xie C. (2020). Improved *cis*-Abienol production through increasing precursor supply in *Escherichia coli*. Sci. Rep..

[21] Yang J.M., Zhao G., Sun Y.Z., Zheng Y.N., Jiang X.L., Liu W., Xian M. (2012). Bio-isoprene production using exogenous MVA pathway and isoprene synthase in *Escherichia coli*. Bioresour. Technol..

[22] Edwards R.A., Keller L.H., Schifferli D.M. (1998). Improved allelic exchange vectors and their use to analyze 987P fimbria gene expression. Gene.

